# A modified Malecot catheter design to prevent complications during difficult percutaneous nephrostomy

**DOI:** 10.1080/2090598X.2019.1626587

**Published:** 2019-06-27

**Authors:** Parag Sonawane, Arvind Ganpule, Sudharsan B, Abhishek Singh, Ravindra Sabnis, Mahesh Desai

**Affiliations:** Department of Urology, Muljibhai Patel Urological Hospital, Nadiad, India

**Keywords:** Malecot catheter, percutaneous nephrostomy, contrast study, guidewire

## Abstract

**Objective**: To demonstrate the feasibility of using a modified Malecot catheter with a proximal end adapter as compared to the conventional Malecot catheter, and demonstrate the technique of performing a contrast study without removing the guidewire (GW).

**Patients and Methods**: A modified 14-F Malecot catheter with a new proximal end connector with one side channel was used for percutaneous nephrostomy (PCN) under fluoroscopy guidance in five patients. The modified Malecot catheter was introduced over the GW under fluoroscopy guidance. The contrast study was done using the side channel of the connector whilst the GW was *in situ* through the main channel. Five senior urologists were asked to assess the modified Malecot catheter by rating their experience on a 5-point Likert scale, which had three items. In a further five patients, the same urologists performed PCN using a conventional Malecot catheter and again rated their experience on the same Likert scale.

**Results**: Total procedure time, fluoroscopy time, and ease of insertion were comparable in the two groups; however, the ease of the contrast study, security of correct positioning, and overall assessment were reported to be better with the modified Malecot catheter.

**Conclusion**: The modified Malecot design allows for easy percutaneous access comparable to the conventional Malecot catheter, with the advantages of ease of performing a contrast study and better security of correct positioning.

**Abbreviations:** GW: guidewire; PCN: percutaneous nephrostomy; PCS: pelvicalyceal system

## Introduction

The Malecot (Stamey) catheter has been routinely used as a self-retaining tube in the drainage of different body fluids, e.g. urine, bile, pus. It was originally described for use in suprapubic cystostomy, which required the use of a needle with the catheter. This made it difficult to insert in small fluid-filled cavities, such as the pelvis of the kidney [1]. A modification employing a flexible introduction system by Rusnak et al. [2] allowed insertion and drainage of even small fluid-filled spaces. Subsequent design modifications, e.g. the flexible stellate and use of dual stiffness material, improved the insertion properties and patient acceptance [1].

Most institutes have standardised protocols for percutaneous nephrostomy (PCN) placement and often the procedure is performed without complications. However, occasionally a difficult PCN can be encountered. Grossly hydronephrotic kidneys with thin parenchyma, obesity, and staghorn calculi are situations that can hinder the safe placement of a PCN tube. When a thick purulent pelvicalyceal system (PCS) collection does not drain easily from the PCN tube, confirming the position necessitates a contrast study that cannot be performed with the guidewire (GW) *in situ*.

The aim of our present study was to assess the feasibility of using a modified Malecot catheter with a proximal end adapter as compared to the conventional Malecot catheter, and demonstrate the technique of performing a contrast study without removing the GW.

## Patients and methods

A modified 14-F polyurethane Malecot catheter with a proximal end adapter was used in this study. The modified Malecot catheter was used for PCN under fluoroscopy guidance in five patients. Five senior urologists were asked to validate the modified Malecot catheter by rating their experience on a 5-point Likert scale, which had three items. The same urologists then performed PCN later in five patients using a conventional Malecot catheter and rated their experience on the same Likert scale.

The data were assessed using the Microsoft Excel 2010, Statistical Package for the Social Sciences (SPSS®) version 10 software (SPSS Inc., Chicago, IL, USA) and the Student’s *t*-test was used to test for significance. Being a pilot study with a small sample size, no attempt was made to assess non-inferiority as compared to the conventional Malecot catheter.

## Results

**Figure 1.**
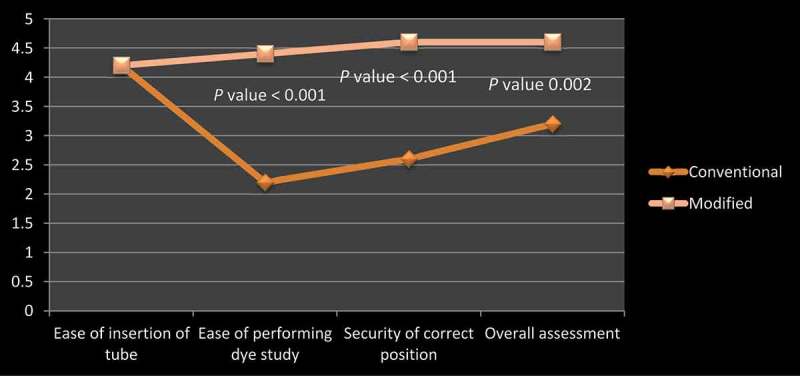
Comparison of Likert scale scores by experts in using the conventional and modified Malecot catheter.

The modified Malecot catheter was successfully placed in all five patients without the need for removal of the GW from the main channel. The total procedure time, fluoroscopy time, and ease of insertion were comparable in the two groups (Table 1); however, the ease of the contrast study, the security of a correct position, and overall assessment were rated better with the modified Malecot catheter (Table 2, Figure 1)

**Table 1.** Intraoperative variables.

Variable, mean (SD)	Conventional Malecot	Modified Malecot	*P*
Fluoroscopy time, s	37.8 (18.7)	49.6 (4.51)	0.20
Total procedure time, min	17.6 (1.82)	17.8 (1.48)	0.85

**Table 2.** Likert scale scores.

Malecot design	Ease of insertion of tube	Ease of performing contrast study	Security of correct position	Overall assessment
Likert score, mean (SD)				
Conventional	4.2 (0.45)	2.2 (0.45)	2.6 (0.55)	3.2 (0.45)
Modified	4.2 (0.45)	4.4 (0.55)	4.6 (0.55)	4.6 (0.55)
*P*	1.0	<0.001	<0.001	0.002

### Design

**Figure 2.**
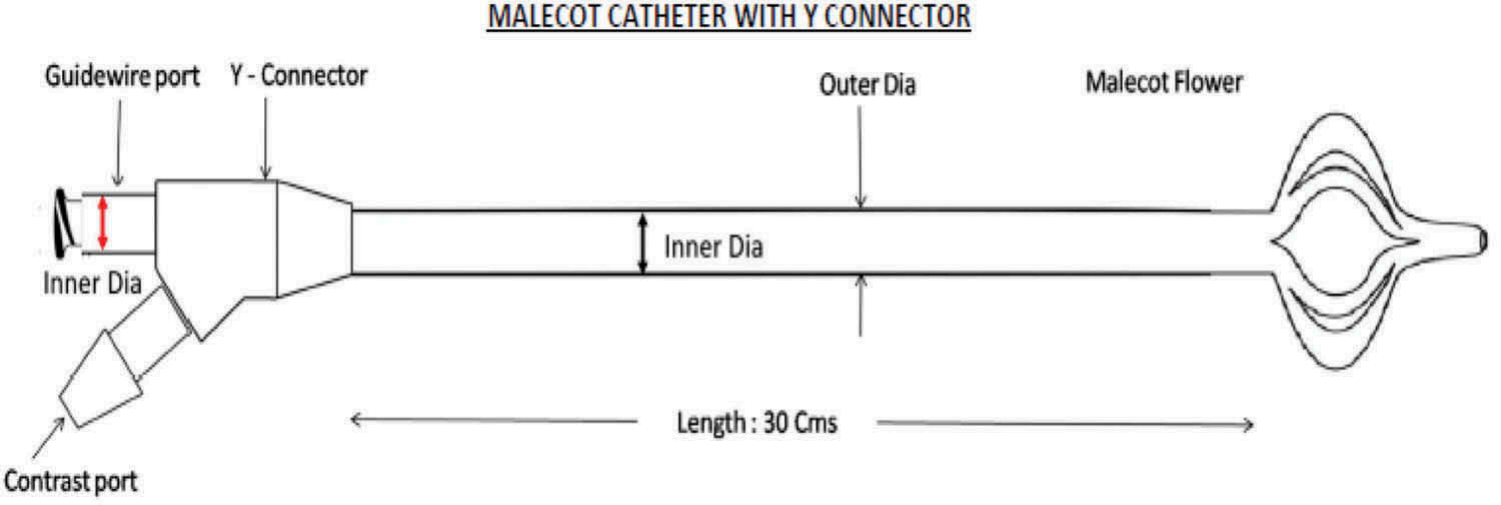
Modified Malecot catheter design.

The proximal end adapter (Y connector) in the modified catheter has the conventional central channel and an offset side channel (contrast port) both opening into a common channel of 7.5 F through the main shaft of the catheter (Figure 2). The distal end mushroom design and the stellate are similar to the conventional Malecot catheter.

The new 14-F Malecot catheter is made of polyurethane and has an outer diameter of 14 F and inner diameter of 7.5 F, whilst the proximal end adapter has a GW port of 7.3 F and a contrast port of 5 F.

### Technique

**Figure 3.**
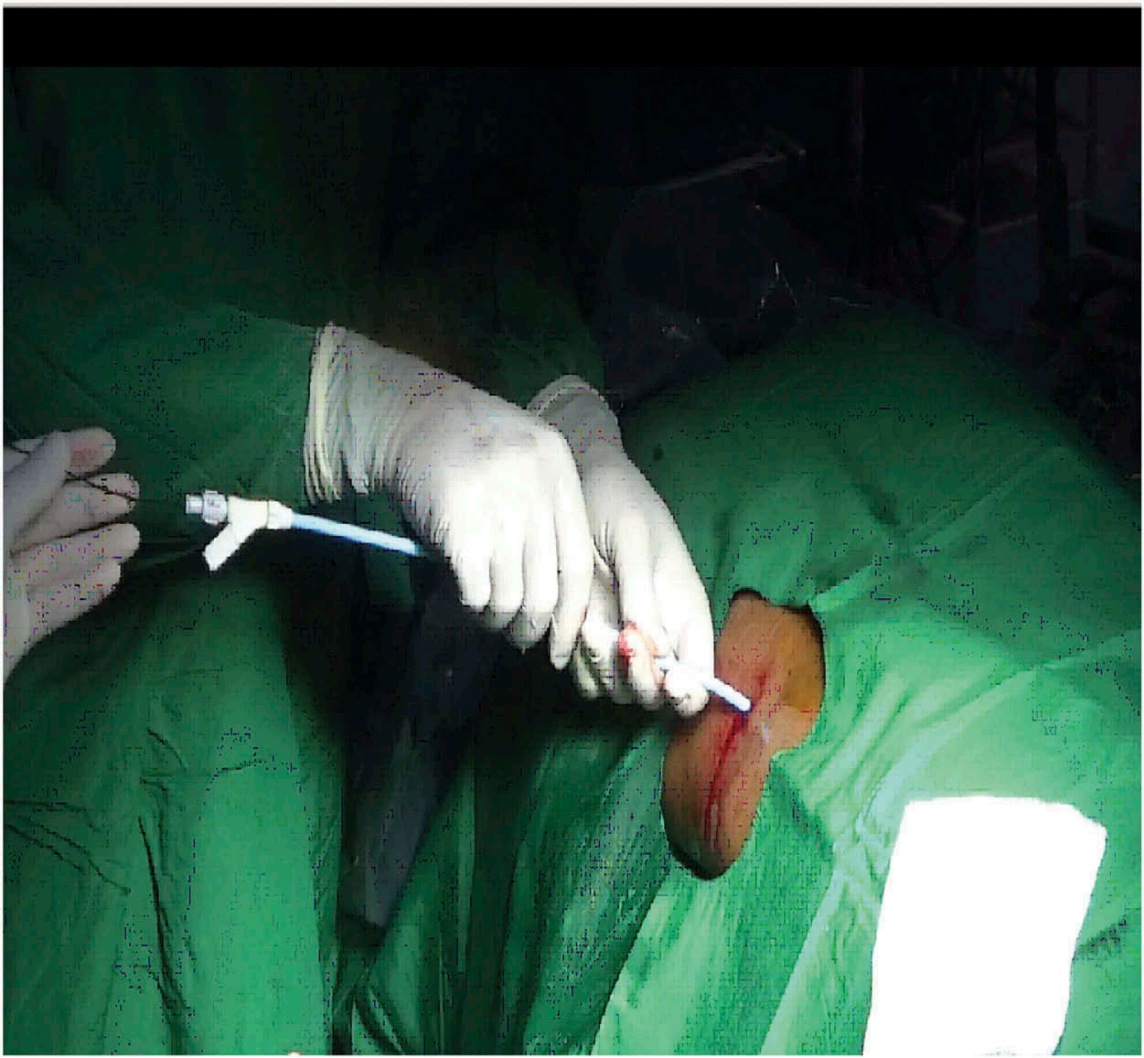
Insertion of Malecot catheter with side channel over GW.

**Figure 4.**
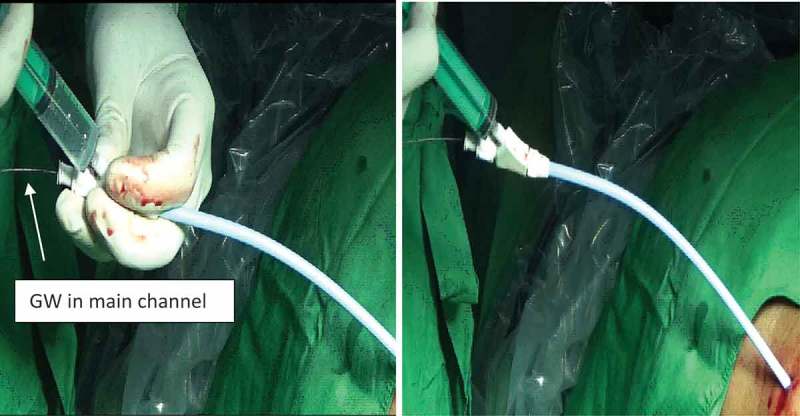
Contrast study via side channel with GW *in situ* in main channel.

**Figure 5.**
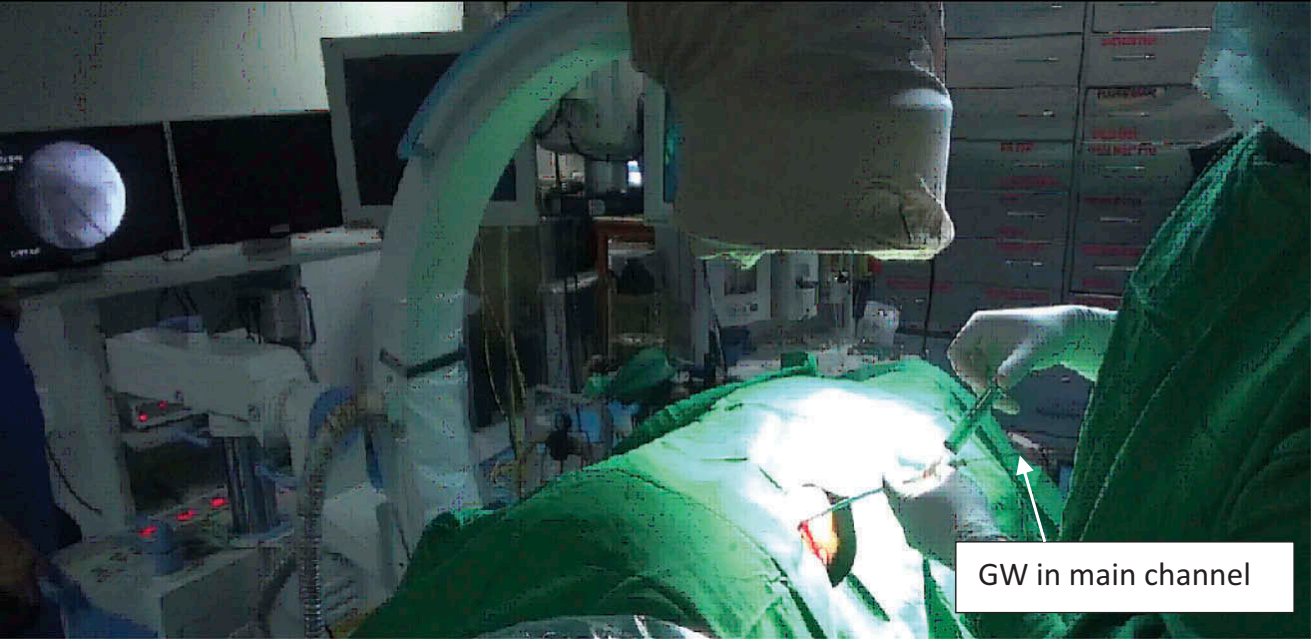
Contrast seen opacifying the PCS (note that GW *in situ*).

Initial percutaneous access is obtained by ultrasound-guided puncture in all cases. Once entry into the PCS through the desired calyx is confirmed, a 0.076 cm (0.03 inch) floppy tip GW is inserted into the PCS and preferably into the ureter. After single-step dilatation, using a screw dilator over the GW, the modified Malecot catheter is introduced over the GW under fluoroscopy guidance (Figure 3). Once the desired position in the PCS is reached, contrast is instilled using the side channel of the proximal end adapter whilst the GW remains *in situ* through the main channel (Figure 4). This allows confirmation of the correct insertion of the Malecot catheter into the PCS, as well as the relative position of the catheter in the PCS (Figure 5). Throughout these steps the GW is still in position through the main channel allowing the repositioning of the catheter whenever incorrect insertion is encountered.

## Discussion

The PCN technique and catheter designs have evolved since its first description in 1955. It can be a temporary or permanent procedure for urinary drainage in obstruction, infection or preoperatively.

Various factors have been described that can predispose to an incorrect placement of the PCN catheter:

1. Inadequate tract dilatation.
2. Obesity, which increases the chances of deviation.
3. Perinephric urinoma resembling dilated calyx due to extravasation of contrast [3].

In addition, we have encountered malpositioning of the PCN catheter when a grossly hydronephrotic kidney with thin parenchymal backing buckles at the time of insertion of the catheter, so that the catheter deviates with sustained pressure and remains outside the PCS. Similarly while inserting a nephrostomy tube in the case of staghorn calculus, the tube can deviate due to a lack of space in the PCS. Problems can also arise when complete access has not been achieved, with the GW coiled up only in the calyx. Often a thick purulent collection may not easily drain out at the outset when the GW is removed from the PCN and confirming the position may need contrast instillation under fluoroscopy.

All these conditions can complicate a simple appearing procedure with significant morbidity. In such situations it would be preferable to be able to confirm the correct position of the nephrostomy tube whilst the well placed GW is still *in situ*.

If the GW is removed and the PCN is found to be out of position, a repeat puncture could become difficult due to collapse of the PCS from the previous puncture, thus reinforcing the need to maintain the previous GW. To overcome these challenges during a difficult PCN placement, we designed a modified Malecot catheter. The newly designed Malecot has the main shaft, stellate and distal mushroom similar to the conventional Malecot (Stamey) catheter. In addition, it incorporates a newly designed proximal end adapter that has two channels. One central main channel and one offset smaller channel opening into the main channel. The inline main channel acts as the drainage channel (GW passes through it), whilst the offset side channel allows the contrast study to be performed without the need to remove the GW from the main channel at the time of PCN placement. As noted in the results, the operating urologists rated the new catheter better in terms of ability to perform the contrast study and for the sense of security. This could be relevant when difficult PCN is anticipated, e.g. in a grossly dilated PCS leading to the risk of slippage, isolated calyceal punctures with wire coiling in the calyx, impacted stones hindering passage of GW into the ureter, and during PCN change.

An alternate practical approach for performing a simultaneous contrast study with the GW *in situ* to a certain extent can be achieved using a tri-way cannula at the proximal end of the Malecot catheter, wherein the GW passes through the straight channel of the tri-way and contrast can be instilled from the side channel. The problem faced in this kind of setup is that most of the contrast leaks by the side of the GW via the straight channel causing variable amounts of contrast to pass into the PCS. Our present Malecot design incorporates this practical approach into a single system and has shown minimal contrast leak with a satisfactory volume of contrast entering the PCS; however, further validation is required.

### Limitations

Although statistically significant in several new aspects, as the present study was a pilot study no comparison could be made with the conventional catheter for non-inferiority or superiority in terms of intraoperative complications of difficult PCN placement. A multi-institutional study with a larger sample size using the modified catheter design is needed to validate the utility of the modified Malecot catheter design.

## Conclusion

The modified Malecot design allows for percutaneous access comparable to the conventional Malecot catheter with the added advantage of ease of performing a contrast study and better security of correct positioning.
